# Preoperative CRP–Albumin–Lymphocyte (CALLY) Index Is Associated with Survival but Does Not Predict Anastomotic Leak or Major Morbidity After Minimally Invasive Esophagectomy

**DOI:** 10.3390/jcm15197567

**Published:** 2026-09-29

**Authors:** Ömer Çağlıyan, Yiğit Düzköylü, Erdal Karaköse, Hüseyin Kılavuz, Hüsnü Ozan Şevik, Oğuzhan Tekin, Ezgi Doğa Yoran, Oğuzhan Hakan Topgül, Orkhan Verdiyev, Zafer Teke, Sercan Yüksel

**Affiliations:** 1 Department of Gastroenterological Surgery, Başakşehir Çam and Sakura City Hospital, 34480 Istanbul, Turkey; dryigit@gmail.com (Y.D.); erdalkarakose71@gmail.com (E.K.); edoa91@hotmail.com (E.D.Y.); naxronic@gmail.com (O.V.); zteke_md@hotmail.com (Z.T.); 2 Department of General Surgery, Başakşehir Çam and Sakura City Hospital, 34480 Istanbul, Turkey; drhuseyinkilavuz@gmail.com (H.K.); drhusnusevik@gmail.com (H.O.Ş.); tekin627@hotmail.com (O.T.); drsercanyuksel@gmail.com (S.Y.)

**Keywords:** esophagectomy, minimally invasive surgery, anastomotic leak, postoperative complications, overall survival, inflammation, nutritional status, CALLY index

## Abstract

**Background/Objectives:** The CRP–albumin–lymphocyte (CALLY) index is promoted both as a marker of postoperative morbidity and as a prognostic factor for survival. These claims are rarely tested together, thresholds usually come from the dataset in which they are then tested, and the index is seldom compared against its components. We examined both in one minimally invasive esophagectomy cohort. **Methods:** Consecutive esophagectomies with a thoracoscopic or robotic thoracic phase at one centre were reviewed. The index was derived from the preoperative albumin, C-reactive protein, and lymphocyte count. Short-term endpoints were major morbidity (Clavien–Dindo grade ≥ 3) and anastomotic leak; survival was analysed by Kaplan–Meier and Cox methods, with internal cut-points stress-tested by bootstrapping. **Results:** Of 154 esophagectomies screened, 22 open or transhiatal procedures and one exploration without resection were excluded, leaving 131 patients. Major morbidity occurred in 39 (29.8%), and leak in 21 (16.0%). The index discriminated neither: area under the curve 0.556 (95% CI: 0.450–0.663) and 0.481 (95% CI: 0.339–0.623). Over a median follow-up of 31.0 months, there were 39 deaths, and median survival was 62.1 months. Here, the index behaved differently: each unit of log-CALLY carried a hazard ratio of 0.776 (95% CI: 0.643–0.936, *p* = 0.008), and three-year survival rose from 48.5% in the lowest tertile to 77.1% in the highest (trend *p* = 0.020). The association held after adjustment for stage and nodal status (0.749, 95% CI: 0.609–0.922, *p* = 0.006), lifting the concordance index of a stage-based model from 0.699 to 0.741. However, entered with CRP, the index lost significance. **Conclusions:** The index said nothing useful about early surgical morbidity yet was independently associated with survival, although that signal did not survive adjustment for CRP alone. It belongs with prognostic markers rather than perioperative risk scores.

## 1. Introduction

Esophagectomy poses two prognostic questions that are usually asked separately. In the first weeks, the question is whether the patient will get through the operation intact: in an international benchmark of high-volume centres, three in five patients sustained a complication and one in six a complication of Clavien–Dindo grade IIIb or worse [1,2], and anastomotic leak, defined by consensus criteria [3] and seen in 11% of resections [2], leads to reoperation, prolonged critical care and early death. Later, the question becomes whether the cancer returns. A single preoperative measurement answering both would be worth having, and indices built from routine blood work are attractive because they cost nothing.

The CALLY index combines serum albumin, absolute lymphocyte count, and C-reactive protein, standing respectively for nutritional reserve, cellular immune competence, and systemic inflammation. Iida and colleagues introduced it in patients undergoing hepatectomy for hepatocellular carcinoma, where lower values marked worse recurrence-free and overall survival [4].

The index has since been studied across most of the gastrointestinal tract. Low values have been linked to poorer survival after pancreaticoduodenectomy for distal cholangiocarcinoma [5], after colorectal resection [6] and after gastrectomy [7,8], and a systematic review of digestive system cancers found the direction of effect consistent between tumour types [9]. In esophageal cancer, several cohorts report that higher values accompany better survival [10,11,12].

More recent literature makes the stronger claim that the index also anticipates postoperative morbidity. In obstructive colorectal cancer treated with a colonic stent, a Japanese multicentre study found a low index to be an independent predictor of complications [13], and in gastric cancer, the index has been tied to major complications and to survival [7]. Two esophageal cohorts report that it predicts postoperative pneumonia [14,15], and low values have been linked to anastomotic complications, both in a 180-patient series reporting a higher leak rate [12] and in a multicentre study of 657 patients at a threshold of 2.55 [16]. On the strength of this combined evidence, the index is increasingly proposed as a general perioperative risk tool.

There are reasons to test that proposal rather than adopt it. The two endpoints answer to different biology: an anastomosis depends on conduit perfusion, tension, and technique, while survival depends on tumour burden and the reserve to tolerate what comes next. Most supporting studies are retrospective and take their threshold from the same dataset in which they then test it, which produces unstable cut-points and optimistically small *p*-values [17]; published CALLY thresholds vary widely, from below two to five [4,14,15,16]. The trials that established minimally invasive esophagectomy also showed a complication profile unlike open surgery [18,19,20], so thresholds drawn from open cohorts cannot be assumed to carry over.

We therefore examined both questions in one consecutive series of minimally invasive esophagectomies, with both endpoints prespecified and an analysis built to expose the fragility of any threshold we might find.

## 2. Materials and Methods

### 2.1. Study Design and Patients

This retrospective cohort study covered consecutive patients who underwent esophagectomy for esophageal or esophagogastric junction cancer in the Department of Gastroenterological Surgery, Başakşehir Çam and Sakura City Hospital, Istanbul, between December 2020 and July 2026. It followed the Declaration of Helsinki and was approved by the hospital’s Clinical Research Ethics Committee No. 2 (approval number KAEK/24.06.2026.348, reference E-96317027-514.10-319251692, dated 2 July 2026). Individual informed consent was waived because the design was retrospective and the data were de-identified. Reporting follows the STROBE recommendations [21].

Patients were eligible if the thoracic phase was completed thoracoscopically or robotically (da Vinci Xi Surgical System; Intuitive Surgical, Sunnyvale, CA, USA). Resections performed through a thoracotomy and transhiatal resections were excluded at the outset, as were patients in whom exploration showed unresectable disease and no resection followed. Where the abdominal phase was open, the operation was classified as hybrid, and these patients were kept in the primary analysis with a prespecified sensitivity analysis confined to totally minimally invasive procedures.

Neoadjuvant treatment followed a uniform institutional protocol. Chemoradiotherapy consisted of weekly carboplatin and paclitaxel with concurrent radiotherapy of 41.4 Gy in 23 fractions, as in the CROSS regimen [22], escalated to 50.4 Gy in selected patients with bulky tumours, and surgery followed six to eight weeks after its completion. For adenocarcinoma, perioperative FLOT was introduced in 2023 and progressively replaced chemoradiotherapy; before and during this transition, patients with adenocarcinoma received the same chemoradiotherapy regimen. Chemotherapy consisted of four preoperative cycles of FLOT (fluorouracil, leucovorin, oxaliplatin, and docetaxel) [23], with surgery three to four weeks after the last cycle. All anastomoses, cervical after McKeown resection and intrathoracic after Ivor Lewis resection, were esophagogastrostomies fashioned with a 25 mm EEA circular stapler with DST Series technology (Covidien, Medtronic, Minneapolis, MN, USA), and construction of the gastric conduit was standardised throughout the study period. All resections were elective. Candidates were reviewed by a multidisciplinary team, and no patient with clinical signs or confirmed evidence of active infection underwent surgery until it had resolved.

### 2.2. Variables and Definitions

Data came from the institutional electronic record and a prospectively maintained departmental database. Blood was drawn at the routine preoperative assessment three days before surgery in every patient, so the interval between sampling and operation was fixed by protocol. Because the interval from the end of neoadjuvant treatment to surgery was also set by protocol, sampling fell at a consistent point after treatment within each regimen. The CALLY index was calculated as albumin (g/L) multiplied by absolute lymphocyte count (×10^3^/µL), divided by the product of C-reactive protein (mg/L) and 10, which is algebraically identical to the original definition in conventional units [4]. The prognostic nutritional index (albumin in g/L plus five times the lymphocyte count) and the CRP-to-albumin ratio were computed from the same samples for comparison.

Complications were graded by the Clavien–Dindo classification [1], retaining each patient’s highest grade, with major morbidity defined as grade 3 or above. Anastomotic leak was recorded when a full-thickness defect of the esophagus, anastomosis, staple line, or conduit was demonstrated, following the Esophagectomy Complications Consensus Group definition [3] and irrespective of how it was found. Leak was not sought by routine imaging. Patients were assessed daily, including chest drain, cervical wound, and drain output, and a suspected leak was confirmed by methylene blue testing, computed tomography with oral and intravenous contrast, or endoscopy. Leaks were managed conservatively where possible, with endoscopic stenting or endoscopic vacuum therapy, drainage, culture-directed broad-spectrum antibiotics, and nutritional support; reoperation was generally avoided. Other short-term endpoints were 30-day mortality, readmission within 30 days, aspiration pneumonia and length of stay. Overall survival, the prespecified long-term endpoint, ran from surgery to death from any cause, including perioperative deaths, with patients not known to have died censored on 20 August 2026.

### 2.3. Statistical Analysis

Continuous variables are given as median (interquartile range) and compared by the Mann–Whitney U test; categorical variables as counts with percentages, compared by chi-square or Fisher’s exact test. The index was strongly right-skewed (skewness 2.13; Shapiro–Wilk *p* < 0.001), so its natural logarithm was used in every regression model.

For the short-term endpoints, we used receiver operating characteristic analysis, estimating areas under the curve and their confidence intervals by the method of DeLong [24], which also served to compare markers measured in the same patients. Whether each area differed from 0.5 was tested by the Mann–Whitney U test, to which that comparison is equivalent. DeLong intervals were checked against 4000 bootstrap replications; for 30-day mortality, with four events, no interval is reported because variance estimates are unreliable with so few events. The optimal cut-point was located by maximising the Youden index. Because such cut-points are unstable, we characterised the sampling variability of the threshold itself with 1000 bootstrap replications, each repeating the whole search, and reported performance at thresholds taken from the literature alongside. Associations were examined by logistic regression, with Firth penalisation where events were sparse.

Survival was estimated by the Kaplan–Meier method with Greenwood standard errors and compared by the log-rank test, with a version for trend across ordered tertiles. Hazard ratios came from Cox regression using the Efron approximation for tied event times, and proportional hazards were checked by correlating scaled Schoenfeld residuals with ranked survival time. Discrimination was measured by Harrell’s concordance index [25], and the added value of the index was assessed by comparing a stage-based model with and without it. Median follow-up was estimated by the reverse Kaplan–Meier method. For illustration, the index was also dichotomised at the cohort median and at the published esophageal threshold of 2.55 [16]; neither cut-point was derived from the survival data.

Because many candidate variables were screened, univariable *p*-values were corrected by the Benjamini–Hochberg false discovery rate procedure [26] and reported next to the uncorrected values. Multivariable models were kept small enough to respect roughly ten events per variable [27]. Analyses used Python 3.12 (Python Software Foundation, Wilmington, DE, USA) with SciPy 1.17 and NumPy 2.4, with a two-sided *p*-value below 0.05 taken as significant.

## 3. Results

### 3.1. Cohort Composition and Baseline Characteristics

One hundred and fifty-four consecutive esophagectomies were screened. Twenty-two were excluded because the resection was performed through a thoracotomy or by a transhiatal approach, leaving 132 with a minimally invasive thoracic phase; one further patient underwent exploration without resection, so 131 entered the analysis (Figure 1). The thoracic phase was robotic in 47 patients and thoracoscopic in 84; the abdominal phase was minimally invasive in 100 and open in 31. Sixty patients had an Ivor Lewis esophagectomy with an intrathoracic anastomosis, and 71 had a McKeown procedure with a cervical anastomosis.

Median age was 60 years (IQR 52–68), and 69 patients (52.7%) were women. Median body mass index was 23.7 kg/m^2^ (IQR 21.2–26.3), missing in 18 patients (13.7%), and 41 patients (31.3%) were ASA class 3 or 4. Squamous cell carcinoma accounted for 72 tumours, adenocarcinoma for 55, and other histologies for four. Neoadjuvant treatment had been given to 123 of the 130 patients with recorded data (94.6%): chemoradiotherapy in 96 (67 squamous cell carcinomas, 25 adenocarcinomas, and four other histologies) and FLOT chemotherapy in 27 (25 adenocarcinomas and two squamous cell carcinomas). Median operative time was 335 min (IQR 300–386) and nodal yield was 20 (IQR 16–31). Median preoperative albumin was 41 g/L (IQR 38–44), CRP 3.0 mg/L (IQR 1.6–7.3), and lymphocyte count 1.41 × 10^3^/µL (IQR 1.00–1.92), giving a median CALLY index of 1.89 (IQR 0.77–3.78). Table 1 shows baseline characteristics by the occurrence of major morbidity.

### 3.2. Short-Term Outcomes and the CALLY Index

Thirty-nine patients (29.8%) sustained a complication of grade 3 or higher and 21 (16.0%) developed an anastomotic leak, of which 18 required intervention and were graded 3 or above. Thirty-day mortality was 3.1% (four patients), readmission within 30 days was 16.0% (21 patients), and aspiration pneumonia was 15.4% (20 patients). Median length of stay was 10 days (IQR 7–15): 8 days (IQR 7–10) without major morbidity and 19 days (IQR 12–40) with it, a difference that reflects the complications themselves.

The index did not identify these patients. Values were lower in those with major morbidity, 1.41 (IQR 0.67–3.00) against 1.96 (IQR 0.80–3.96), but the difference fell short of significance (*p* = 0.310); the effect was small (rank-biserial correlation −0.11), and the area under the curve was 0.556 (95% CI: 0.450–0.663). For leak, the index carried no information at all: 1.69 (IQR 0.90–5.59) against 1.89 (IQR 0.77–3.48) (*p* = 0.785), area under the curve 0.481 (95% CI: 0.339–0.623). Figure 2 and Figure 3 show the distributions and curves.

As a continuous variable, the index predicted neither endpoint (major morbidity: OR 0.94 per unit, 95% CI: 0.85–1.04, *p* = 0.254; leak: OR 1.02, 95% CI: 0.91–1.13, *p* = 0.780), and spline modelling gave no sign of a threshold or U-shaped relationship (departure from linearity *p* = 0.464 and *p* = 0.935). Across tertiles, major morbidity fell only slightly, from 34.1% to 27.3% (trend *p* = 0.488), and for leak, there was no gradient at all (15.9%, 14.0%, 18.2%; trend *p* = 0.773). Table 2 summarises these results.

The threshold maximising the Youden index was 1.563, giving 56.4% sensitivity and 62.0% specificity for major morbidity, yet even at this internally optimised value the association fell short of significance (OR 2.11, 95% CI: 0.99–4.51, *p* = 0.055). Bootstrap resampling placed the 95% confidence interval of the cut-point itself between 0.74 and 6.86, a ninefold range. At 2.55, the threshold from the largest published esophageal cohort, the association was weaker still (OR 1.29, 95% CI: 0.59–2.82, *p* = 0.531). Table 3 gives performance across thresholds.

No competing marker performed better. For major morbidity, the areas under the curve were 0.614 for body mass index, 0.599 for operative time, 0.578 for albumin, 0.570 for the CRP-to-albumin ratio, 0.559 for CRP, 0.556 for the CALLY index, and 0.543 for the prognostic nutritional index. None reached significance, nor did any pairwise DeLong comparison against the index (*p* ≥ 0.10), and for leak, every marker sat near 0.5. On univariable analysis, major morbidity was associated with female sex (OR 0.38, *p* = 0.014) and squamous histology (OR 0.45, *p* = 0.039), and leak with the hybrid approach (OR 3.00, *p* = 0.028), McKeown reconstruction (OR 0.36, *p* = 0.041), and squamous histology (OR 0.27, *p* = 0.011). None survived correction for multiplicity; the smallest adjusted q-values were 0.286 and 0.229.

### 3.3. Overall Survival and the CALLY Index

Median follow-up was 31.0 months (range 0.4–67.0), with 39 deaths. Median overall survival was 62.1 months: 79.7% (95% CI: 72.6–86.9) survived one year, 72.0% (95% CI: 63.6–80.5) two years, and 66.1% (95% CI: 56.5–75.6) three years.

Against the short-term picture, the index tracked survival. Each unit of the log-transformed index carried a hazard ratio of 0.776 (95% CI: 0.643–0.936, *p* = 0.008) or 0.686 (95% CI: 0.519–0.906) per standard deviation, with a concordance index of 0.635. The proportional hazards assumption held (Schoenfeld correlation 0.064, *p* = 0.701).

The relationship was graded across tertiles (Figure 4). Three-year survival was 48.5% in the lowest tertile, 74.2% in the middle, and 77.1% in the highest, with median survival of 32.3 months versus 62.1 months between the extremes (log-rank *p* = 0.030; trend *p* = 0.020). Against the highest tertile, the lowest carried a hazard ratio of 2.31 (95% CI: 1.08–4.95, *p* = 0.031), while the middle tertile did not differ (1.09, *p* = 0.849). Split at the cohort median of 1.89, a value not derived from the survival data, the curves separated similarly: median survival 54.3 against 62.1 months, three-year survival 55.1% against 77.3%, hazard ratio 2.00 (95% CI: 1.04–3.86, log-rank *p* = 0.034). At the published esophageal threshold of 2.55 [16], the difference ran in the same direction but did not reach significance (three-year survival 58.7% against 79.2%; hazard ratio 1.95, 95% CI: 0.95–4.02; log-rank *p* = 0.063).

In the univariable Cox analysis, the strongest associations with death were anastomotic leak (hazard ratio 5.42, 95% CI: 2.71–10.85), metastatic nodes (1.14 per node), major morbidity (3.88), T3–T4 stage (3.94), and nodal involvement (3.67). CRP (1.03 per mg/L), the CRP-to-albumin ratio (1.09), the hybrid approach (2.90), squamous histology (0.38), the log-CALLY index (0.78), and albumin (0.93) were also associated. Eleven variables survived correction for multiplicity; the index among them (q = 0.017; Table 4).

The index kept its independence in multivariable models. Adjusted for tumour stage, nodal status and age, it carried a hazard ratio of 0.749 (95% CI: 0.609–0.922, *p* = 0.006), alongside T3–T4 stage (3.02) and nodal involvement (2.45), with a concordance index of 0.741. In a second model replacing stage with the postoperative course, it remained significant (0.788, 95% CI: 0.645–0.963, *p* = 0.020) alongside nodal involvement (3.13) and major morbidity (4.70), giving a concordance index of 0.778 with no violation of proportional hazards. Adding the index to a model holding T stage and nodal status alone raised the concordance index from 0.699 to 0.741. Table 5 shows both models. Survival estimates by tertile and by cut-point are given in Table 6.

Perioperative deaths did not drive the result: restricting the analysis to the 117 patients surviving beyond 90 days left the hazard ratio almost unchanged (0.751, 95% CI: 0.608–0.927, *p* = 0.008). The association was also present in the 100 totally minimally invasive procedures (0.63, *p* < 0.001), in squamous tumours (0.74, *p* = 0.032), and in node-positive disease (0.69, *p* = 0.010), though not in the Ivor Lewis subgroup (0.89, *p* = 0.368) or in node-negative patients (0.86, *p* = 0.344), where deaths numbered 20 and 15, respectively.

One result qualifies the interpretation. Entered together with CRP, the index lost significance (hazard ratio 0.93, *p* = 0.610) while CRP kept a borderline association (1.02 per mg/L, *p* = 0.068); CRP alone carried a hazard ratio of 1.027 (95% CI: 1.012–1.043, *p* < 0.001). In this cohort, the index was almost perfectly collinear with the reciprocal of CRP (Spearman ρ = −0.93) and only weakly related to albumin (ρ = 0.24).

## 4. Discussion

In the same 131 patients, and from the same blood sample, the CALLY index told us nothing useful about early surgical morbidity yet remained independently associated with survival. For anastomotic leak, the area under the curve was 0.481, which is chance; for death, the adjusted hazard ratio was 0.749 per unit of the log-transformed index, surviving both correction for multiplicity and adjustment for stage. That split is the main observation here, and it says more than either half alone.

The split makes biological sense. Anastomotic leak after minimally invasive esophagectomy depends on conduit perfusion, tension, and technical execution, none of which a peripheral blood sample can measure. In this series, the stapling technique was uniform: a 25 mm circular esophagogastrostomy on a standardised gastric conduit, so variation in leak lay in the level of the anastomosis, the operative approach, and the patient rather than in the method of anastomosis. The strongest signals for leak in our data were structural rather than serological, and even those dissolved once confounding by indication was taken into account: the apparent protection offered by cervical reconstruction ran against the usual direction of report and disappeared when histology entered the model. Survival reflects tumour burden and the reserve to tolerate recurrence and further treatment, closer to what an inflammatory and nutritional index might capture. Where an inflammatory marker does flag a leak, the measurement is postoperative rather than preoperative: in a prospective series of minimally invasive esophagectomies, CRP on the fifth postoperative day reached an area under the curve of 0.825 with a negative predictive value of 97% [28].

Our survival data sit comfortably with the wider literature. Across hepatocellular, cholangiocellular, colorectal, gastric, and esophageal cancer, a low index has been repeatedly associated with worse survival [4,5,6,7,8,9,10,11,12], and a systematic review of digestive system cancers found the direction consistent between tumour types [9]; a review of systemic inflammatory markers in esophageal cancer reached the same conclusion for this disease [29]. What we could not reproduce is the morbidity claim. The reports behind it come from settings unlike ours [7,12,13,14,15,16], including obstructive colorectal cancer requiring a stent, gastrectomy and esophagectomy in largely treatment-naive patients, and the endpoint has usually been pneumonia or a composite rather than anastomotic leak. A single cohort cannot settle whether the difference lies in the endpoint, the population, or the handling of thresholds, but the claim does not carry over to minimally invasive esophagectomy after neoadjuvant therapy.

Threshold selection deserves separate comment because it bears on the wider literature rather than on our cohort alone. Our Youden threshold of 1.563 had a bootstrap confidence interval running from 0.74 to 6.86, and the association at that value did not reach significance. Published CALLY thresholds range from below two to five [4,14,15,16], and the variation is not confined to different diseases: two cohorts of McKeown esophagectomy for squamous carcinoma, from the same institution and with the same endpoint of postoperative pneumonia, arrived at thresholds of 1.97 and 3.47 [14,15]. Heterogeneity on that scale points to the method of selection at least as much as to biology. Our survival finding does not rest on a threshold at all: the association was continuous and graded across tertiles. Dichotomised at the cohort median, the curves separated, whereas at the published esophageal threshold of 2.55, the difference ran in the same direction without reaching significance, a reminder of how much a single cut-point can hide or suggest.

The population may matter, too. Almost all our patients (94.6%) received neoadjuvant therapy, against about one in ten in the multicentre cohort that linked the index to anastomotic complications [16]. In heavily pretreated patients, preoperative CRP is as likely to reflect treatment-related inflammation as any durable vulnerability, which would blunt a short-term signal while leaving a longer-term one intact. Our sampling was at least consistent, taken three days before surgery in every patient, so the variation we measured is not an artefact of ragged timing. The interval from the end of neoadjuvant treatment was also fixed by protocol, six to eight weeks after chemoradiotherapy and three to four weeks after FLOT. Treatment type left a visible trace: lymphocyte counts were lower after chemoradiotherapy than after chemotherapy (median 1.19 against 1.91 × 10^3^/µL, *p* < 0.001), consistent with radiation-induced lymphopenia. The survival association nevertheless held within each regimen, with a hazard ratio of 0.80 (95% CI: 0.65–0.99, *p* = 0.035) after chemoradiotherapy and 0.51 (95% CI: 0.27–0.96, *p* = 0.038) after FLOT, the latter resting on six deaths, and after adjustment for regimen (0.78, 95% CI: 0.64–0.94, *p* = 0.010). The index is therefore not simply a marker of which treatment a patient received. Median albumin here was 41 g/L, so the nutritional component had little variation to work with.

What the index measures here is worth stating plainly. Entered alongside CRP, it lost significance, and CRP alone was strongly associated with survival. Since CRP sits in the denominator and varies over a far wider range than albumin or lymphocyte count, the composite was nearly collinear with its reciprocal. We cannot claim, then, that the three-component index beats its most volatile constituent. What the data support is that the inflammatory axis CRP captures, however packaged, carries prognostic information for survival but not for anastomotic healing. Studies proposing composite indices should be asked to show value beyond their components.

One secondary result deserves mention. Anastomotic leak carried more than a fivefold hazard of death (5.42, 95% CI: 2.71–10.85), which held in a landmark analysis restricted to patients surviving beyond 90 days (5.82, 95% CI: 2.59–13.04), as did major morbidity (3.09). That early complications carry a long-term oncological cost is not new, but it sharpens the problem: the complication that does most damage to long-term outcome is the one no preoperative blood marker here could see coming.

Two subgroups did not show the association, and both invite a cautious reading. Among node-negative patients and among those having an Ivor Lewis resection, the point estimates ran in the same direction as the whole cohort (0.86 and 0.89), but the confidence intervals crossed unity. With 15 and 20 deaths, these analyses are underpowered, so a type II error is the simpler explanation. A biological account is nonetheless available for the nodal split: in node-negative disease the absolute risk of cancer death is lower and the competing hazards fewer, leaving less variation for a host marker to explain, whereas in node-positive disease the reserve to tolerate recurrence and further treatment matters more. Separating the two would need a much larger cohort, and we present these analyses as exploratory rather than as evidence of effect modification.

The limitations are plain. The study is retrospective and single-centre, and the operative approach shifted over the period, with robotic procedures concentrated early and hybrid procedures commoner later, so technique and era cannot be separated. Median follow-up was 31.0 months with 39 deaths, so estimates beyond three years rest on small numbers and the confidence intervals are wide. The long-term endpoint was all-cause mortality, so cancer and non-cancer deaths are not distinguished. Body mass index was missing in 13.7% of patients, and neutrophil and platelet counts were not consistently available, which ruled out comparison with the neutrophil-to-lymphocyte and platelet-to-lymphocyte ratios and the systemic immune-inflammation index. Sampling and the interval from neoadjuvant treatment were fixed by protocol rather than recorded for each patient, and residual treatment-related inflammation cannot be excluded. Medications that alter CRP or lymphocyte counts, such as corticosteroids, were not systematically recorded. Leak was diagnosed on clinical suspicion rather than by routine imaging, so small subclinical leaks may have been missed, and the endpoint reflects clinically relevant leaks. Complications were graded from the record rather than collected prospectively. With 21 leaks and 39 deaths, the cohort cannot support large multivariable models, and the subgroup analyses are exploratory.

The strengths lie in how the analysis was built. Both classes of endpoint were measured in the same patients from the same sample, which is what makes the split visible at all, and the cohort is homogeneous for the surgical approach. Multiplicity was handled explicitly, the derived threshold was tested rather than assumed, and the added value over stage was measured rather than asserted.

For practice, the index should not be used to triage patients for perioperative risk, to change anastomotic technique, or to defer surgery on the expectation of a leak. It may contribute to prognostic discussion and to stratification in trials, where its addition to stage improved discrimination here. Before it earns that role, prospective studies will be needed with standardised sampling, externally specified thresholds and an explicit comparison against CRP alone.

## 5. Conclusions

Among 131 patients undergoing esophagectomy with a minimally invasive thoracic phase, the preoperative CALLY index did not predict major morbidity or anastomotic leak, and no threshold, including one optimised within the data, gave useful discrimination. The same index was independently associated with overall survival and improved the discrimination of a stage-based model. It appears to track host biology relevant to cancer outcome rather than the determinants of anastomotic healing and is better placed as a prognostic marker than as a perioperative risk score.

## Figures and Tables

**Figure 1:**
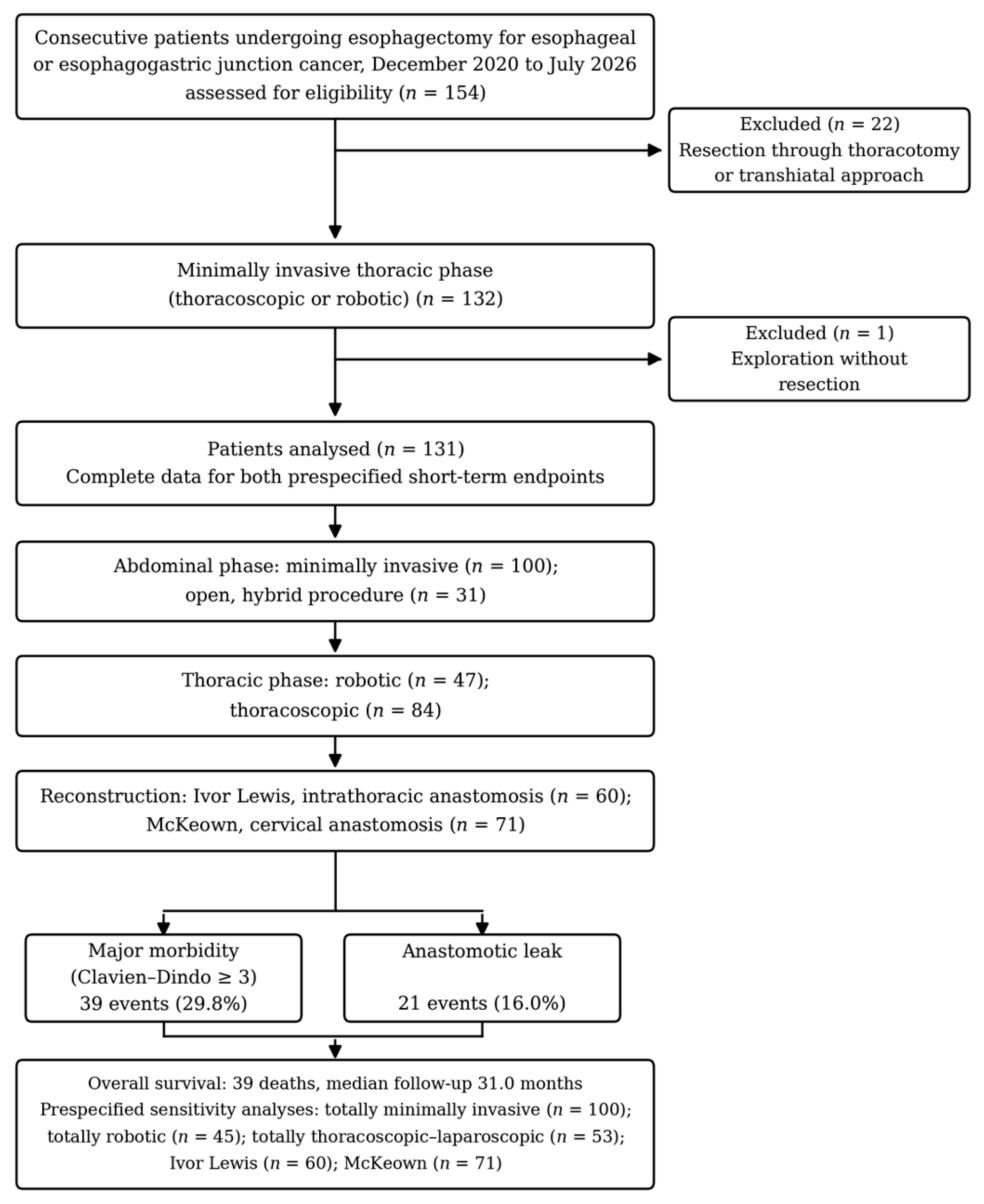
Flow of patients through the study. Of 154 consecutive esophagectomies screened, 22 were excluded because the resection was performed through a thoracotomy or by a transhiatal approach, and one because exploration was not followed by resection, leaving 131 patients.

**Figure 2:**
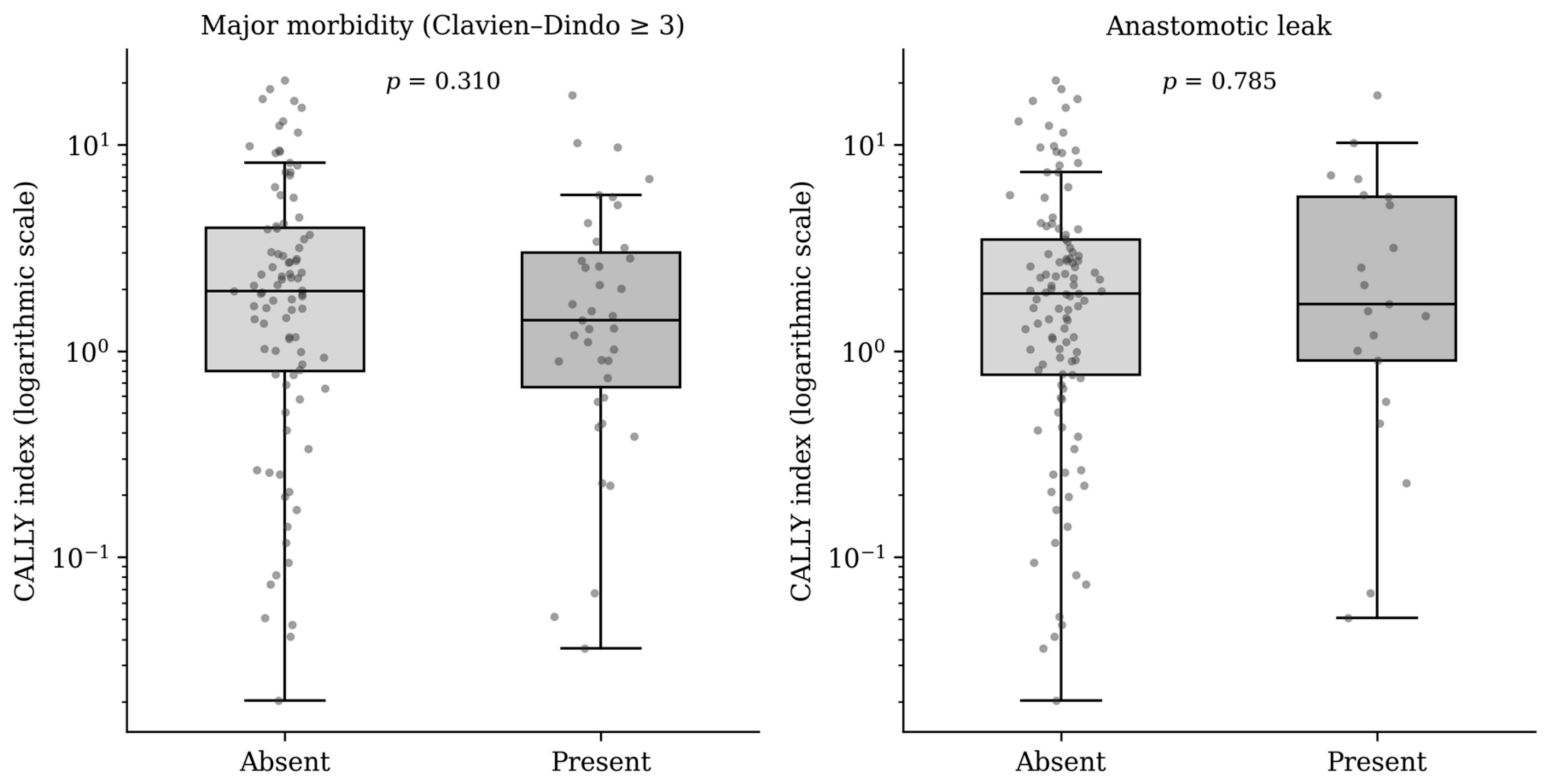
Distribution of the preoperative CALLY index according to the occurrence of major morbidity (Clavien–Dindo grade ≥ 3) and anastomotic leak. Boxes show the median and interquartile range with individual values overlaid; whiskers extend to the most extreme values within 1.5 times the interquartile range; the vertical axis is logarithmic. Neither comparison reached significance (Mann–Whitney U test).

**Figure 3:**
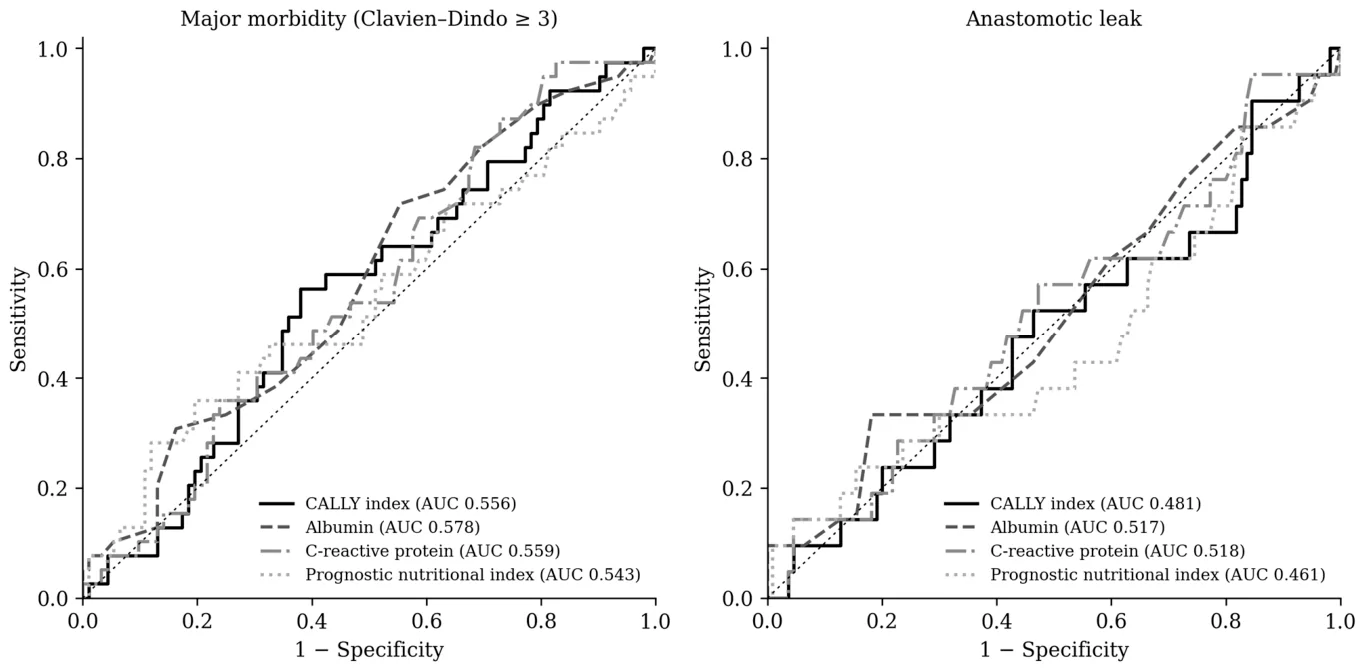
Receiver operating characteristic curves for the preoperative CALLY index, albumin, C-reactive protein and the prognostic nutritional index, for major morbidity (**left**) and anastomotic leak (**right**). All curves lie close to the diagonal (the line of no discrimination, area under the curve 0.5), and no pairwise DeLong comparison reached significance.

**Figure 4:**
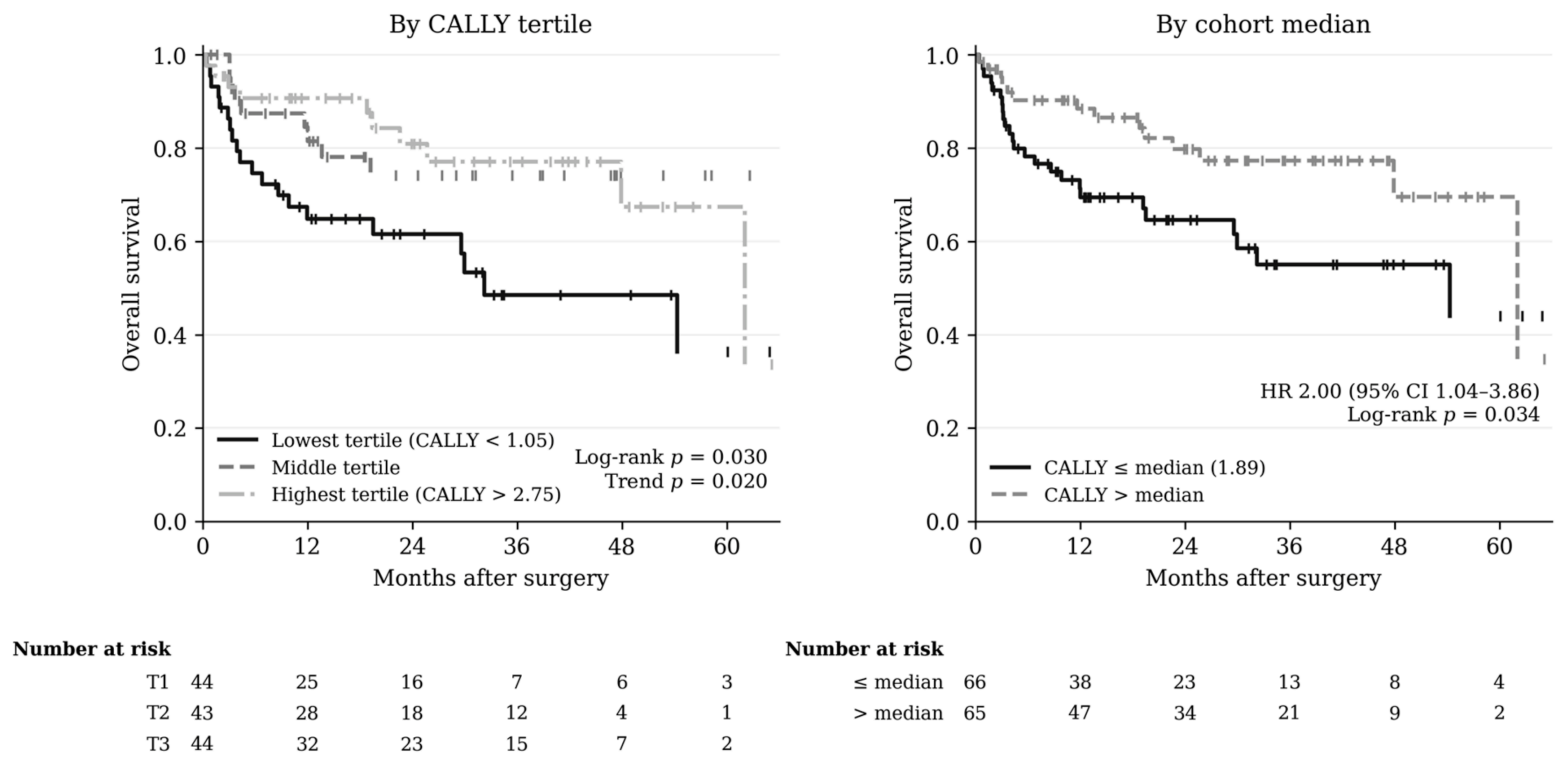
Kaplan–Meier estimates of overall survival according to the preoperative CALLY index, by tertile (**left**) and dichotomised at the cohort median of 1.89 (**right**). Vertical ticks denote censored observations and the numbers at risk are shown beneath each panel.

**Table 1:** Baseline, operative and laboratory characteristics of the study cohort, stratified by the occurrence of major morbidity.

Characteristic	All Patients (*n* = 131)	No Major Morbidity (*n* = 92)	Major Morbidity (*n* = 39)	*p*
*Demographics and comorbidity*				
Age, years	60 (52–68)	60 (53–68)	58 (48–66)	0.234
Female sex	69 (52.7)	55 (59.8)	14 (35.9)	0.021
Body mass index, kg/m^2^	23.7 (21.2–26.3)	24.2 (22.3–26.8)	21.6 (19.4–25.7)	0.060
ASA class ≥ 3	41 (31.3)	29 (31.5)	12 (30.8)	1.000
Diabetes mellitus	27 (21.3)	22 (24.2)	5 (13.9)	0.300
Cardiac disease	36 (28.3)	24 (26.4)	12 (33.3)	0.571
Ever smoker	41 (32.3)	31 (34.1)	10 (27.8)	0.637
*Tumour and treatment*				
Squamous cell carcinoma	72 (55.0)	56 (60.9)	16 (41.0)	0.058
Neoadjuvant therapy	123 (94.6)	84 (92.3)	39 (100.0)	0.102
Nodal involvement (N+)	52 (40.3)	32 (35.6)	20 (51.3)	0.140
*Operative details*				
Robotic thoracic phase	47 (35.9)	34 (37.0)	13 (33.3)	0.844
Hybrid (open abdomen)	31 (23.7)	19 (20.7)	12 (30.8)	0.307
McKeown procedure	71 (54.2)	52 (56.5)	19 (48.7)	0.530
Feeding jejunostomy	10 (7.6)	8 (8.7)	2 (5.1)	0.722
Operative time, min	335 (300–386)	330 (289–376)	350 (310–435)	0.074
Nodes retrieved	20 (16–31)	22 (16–32)	20 (15–28)	0.077
*Preoperative laboratory values*				
Albumin, g/L	41 (38–44)	41 (39–44)	41 (37–42)	0.157
C-reactive protein, mg/L	3.0 (1.6–7.3)	3.0 (1.3–6.1)	3.6 (1.9–9.2)	0.287
Lymphocytes, ×10^3^/µL	1.41 (1.00–1.92)	1.35 (1.01–1.85)	1.42 (1.00–2.08)	0.435
Prognostic nutritional index	48.3 (44.2–52.6)	48.3 (44.5–52.5)	48.0 (42.0–52.4)	0.444
CRP-to-albumin ratio	0.74 (0.38–1.69)	0.73 (0.34–1.51)	0.98 (0.46–2.22)	0.208
CALLY index	1.89 (0.77–3.78)	1.96 (0.80–3.96)	1.41 (0.67–3.00)	0.310

Values are median (interquartile range) or *n* (%). Percentages for variables with missing data are calculated on available cases; body mass index was missing in 18 patients, diabetes, cardiac disease and smoking status in 4 each, nodal status in 2, and neoadjuvant treatment in 1. ASA, American Society of Anesthesiologists; CALLY, C-reactive protein–albumin–lymphocyte; CRP, C-reactive protein. Length of stay, a consequence rather than a determinant of major morbidity, is reported with the postoperative outcomes in Section 3.2.

**Table 2:** Distribution of the preoperative CALLY index by outcome, and discriminative performance.

Outcome	Events, *n* (%)	CALLY, Event Present, Median (IQR)	CALLY, Event Absent, Median (IQR)	*p*	AUC (95% CI)
Major morbidity (CD ≥ 3)	39 (29.8)	1.41 (0.67–3.00)	1.96 (0.80–3.96)	0.310	0.556 (0.450–0.663)
Anastomotic leak	21 (16.0)	1.69 (0.90–5.59)	1.89 (0.77–3.48)	0.785	0.481 (0.339–0.623)
30-day mortality	4 (3.1)	0.66 (0.41–0.90)	1.93 (0.79–3.92)	0.055	0.781 (not estimated)
Readmission within 30 days	21 (16.0)	1.01 (0.26–2.75)	1.94 (0.87–4.02)	0.145	0.601 (0.472–0.730)
Aspiration pneumonia	20 (15.4)	1.49 (0.77–5.91)	1.89 (0.77–3.48)	0.972	0.497 (0.346–0.649)
*Event rate by CALLY tertile*					
T1, median 0.40 (*n* = 44)	—	—	—	—	CD ≥ 3: 34.1%; leak: 15.9%
T2, median 1.89 (*n* = 43)	—	—	—	—	CD ≥ 3: 27.9%; leak: 14.0%
T3, median 5.97 (*n* = 44)	—	—	—	—	CD ≥ 3: 27.3%; leak: 18.2%
Test for trend	—	—	—	—	CD ≥ 3: *p* = 0.488; leak: *p* = 0.773

AUC values assume that a lower CALLY index indicates higher risk. The *p*-values are from the Mann–Whitney U test, which is equivalent to testing whether the AUC differs from 0.5. Confidence intervals are from the DeLong method and agreed with 4000 bootstrap replications for every outcome with at least 20 events. The estimate for 30-day mortality rests on four events, all with CALLY values between 0.38 and 0.90, so no confidence interval is given, and the estimate should not be read as evidence of discrimination. AUC, area under the receiver operating characteristic curve; CI, confidence interval; IQR, interquartile range.

**Table 3:** Diagnostic performance of the CALLY index at internally derived and externally specified cut-points.

Threshold	Source	Sens., %	Spec., %	PPV, %	NPV, %	Odds Ratio (95% CI)
*Major morbidity (CD ≥ 3)*						
1.563	Youden (internal)	56.4	62.0	38.6	77.0	2.11 (0.99–4.51); *p* = 0.055
1.00	Fixed	35.9	70.7	34.1	72.2	1.35 (0.61–2.98); *p* = 0.461
2.00	Fixed	59.0	48.9	32.9	73.8	1.38 (0.65–2.94); *p* = 0.409
2.55	Published (esophageal)	66.7	39.1	31.7	73.5	1.29 (0.59–2.82); *p* = 0.531
3.00	Fixed	74.4	31.5	31.5	74.4	1.33 (0.57–3.10); *p* = 0.502
*Anastomotic leak*						
1.694	Youden (internal)	52.4	53.6	17.7	85.5	1.27 (0.50–3.24); *p* = 0.613
2.55	Published (esophageal)	61.9	37.3	15.9	83.7	0.97 (0.37–2.53); *p* = 0.943

The internally derived threshold was obtained by maximising the Youden index within this dataset; its bootstrap 95% confidence interval was 0.74 to 6.86 for major morbidity and 0.05 to 17.39 for anastomotic leak. Odds ratios are from logistic regression with the index dichotomised at the stated threshold. PPV, positive predictive value; NPV, negative predictive value.

**Table 4:** Univariable Cox regression for overall survival.

Variable	Hazard Ratio (95% CI)	*p*	q (FDR-Adjusted)	Significant
*Significant after correction for multiplicity*				
Anastomotic leak	5.42 (2.71–10.85)	<0.001	<0.001	Yes
Metastatic nodes, per node	1.14 (1.08–1.21)	<0.001	<0.001	Yes
Major morbidity (CD ≥ 3)	3.88 (2.04–7.37)	<0.001	<0.001	Yes
Tumour stage T3–T4	3.94 (2.03–7.65)	<0.001	<0.001	Yes
Nodal involvement (N+)	3.67 (1.91–7.04)	<0.001	<0.001	Yes
C-reactive protein, per mg/L	1.03 (1.01–1.04)	<0.001	0.001	Yes
CRP-to-albumin ratio, per unit	1.09 (1.04–1.14)	<0.001	0.001	Yes
Hybrid approach (open abdomen)	2.90 (1.47–5.73)	0.002	0.006	Yes
Squamous histology	0.38 (0.20–0.73)	0.004	0.008	Yes
log-CALLY index, per unit	0.78 (0.64–0.94)	0.008	0.017	Yes
Albumin, per g/L	0.93 (0.87–0.98)	0.015	0.028	Yes
*Not significant after correction*				
Female sex	0.50 (0.26–0.95)	0.034	0.059	No
McKeown procedure	0.65 (0.35–1.22)	0.181	0.292	No
Body mass index, per kg/m^2^	0.95 (0.87–1.04)	0.258	0.361	No
Operative time, per min	1.00 (1.00–1.01)	0.267	0.361	No
Prognostic nutritional index, per unit	0.97 (0.93–1.02)	0.275	0.361	No
Lymphocytes, per ×10^3^/µL	1.21 (0.82–1.80)	0.335	0.414	No
Robotic thoracic phase	0.76 (0.38–1.49)	0.418	0.475	No
Nodes retrieved, per node	0.99 (0.96–1.02)	0.430	0.475	No
ASA class, per grade	1.15 (0.69–1.93)	0.587	0.617	No
Age, per year	1.01 (0.98–1.04)	0.634	0.634	No

Twenty-one candidate variables were screened. Hazard ratios for continuous variables refer to a one-unit increase. The q-value is the Benjamini–Hochberg adjusted *p*-value across all 21 comparisons. Major morbidity and anastomotic leak are postoperative variables and are reported as prognostic factors rather than baseline predictors; a landmark analysis restricted to patients surviving beyond 90 days gave hazard ratios of 5.82 (95% CI: 2.59–13.04) and 3.09 (95% CI: 1.48–6.47), respectively. CD, Clavien–Dindo; CRP, C-reactive protein.

**Table 5:** Multivariable Cox regression models for overall survival.

Variable	Adjusted Hazard Ratio (95% CI)	*p*	Model Performance
*Model A: adjusted for tumour stage (n = 129, 39 deaths)*			
log-CALLY index, per unit	0.749 (0.609–0.922)	0.006	LR χ^2^ = 31.3 (df 4), *p* < 0.001
Tumour stage T3–T4	3.02 (1.48–6.17)	0.002	Concordance index 0.741
Nodal involvement (N+)	2.45 (1.22–4.92)	0.012	
Age, per year	1.01 (0.98–1.03)	0.572	
*Model B: adjusted for postoperative course (n = 129, 39 deaths)*			
log-CALLY index, per unit	0.788 (0.645–0.963)	0.020	LR χ^2^ = 38.6 (df 4), *p* < 0.001
Nodal involvement (N+)	3.13 (1.60–6.13)	<0.001	Concordance index 0.778
Major morbidity (CD ≥ 3)	4.70 (2.28–9.71)	<0.001	
Age, per year	1.03 (1.00–1.06)	0.048	

Model size was constrained to respect approximately ten events per variable. Model A adjusts the index for tumour stage; Model B replaces stage with the postoperative course. Adding the log-CALLY index to a model containing T stage and nodal status alone raised the concordance index from 0.699 to 0.741. LR, likelihood ratio.

**Table 6:** Overall survival according to the preoperative CALLY index.

Group	*n*	Deaths, *n* (%)	Median Survival, Months	2-Year Survival, %	3-Year Survival, %
*By CALLY tertile*					
Lowest (0.02–1.03)	44	20 (45.5)	32.3	61.6	48.5
Middle (1.11–2.75)	43	9 (20.9)	Not reached	74.2	74.2
Highest (2.75–20.54)	44	10 (22.7)	62.1	80.9	77.1
*By cohort median (1.89)*					
CALLY ≤ 1.89	66	25 (37.9)	54.3	64.7	55.1
CALLY > 1.89	65	14 (21.5)	62.1	79.8	77.3
*By published esophageal threshold (2.55)*					
CALLY ≤ 2.55	82	29 (35.4)	Not reached	65.9	58.7
CALLY > 2.55	49	10 (20.4)	62.1	82.6	79.2
*Whole cohort*					
All patients	131	39 (29.8)	62.1	72.0	66.1

Survival estimates are Kaplan–Meier probabilities. Comparison across tertiles gave a log-rank *p* of 0.030 and a test for trend *p* of 0.020; the hazard ratio for the lowest against the highest tertile was 2.31 (95% CI: 1.08–4.95, *p* = 0.031). Dichotomised at the cohort median, the hazard ratio was 2.00 (95% CI: 1.04–3.86, log-rank *p* = 0.034), and at the published threshold of 2.55, it was 1.95 (95% CI: 0.95–4.02, log-rank *p* = 0.063). Median follow-up was 31.0 months.

## Data Availability

The de-identified dataset analysed during this study is available from the corresponding author on reasonable request. The data are not publicly deposited because they derive from hospital records and remain subject to institutional privacy restrictions.

## Abbreviations

The following abbreviations are used in this manuscript:

ASA	American Society of Anesthesiologists
AUC	Area under the receiver operating characteristic curve
CALLY	C-reactive protein–albumin–lymphocyte index
CD	Clavien–Dindo
CI	Confidence interval
CRP	C-reactive protein
ECCG	Esophagectomy Complications Consensus Group
FDR	False discovery rate
FLOT	Fluorouracil, leucovorin, oxaliplatin, and docetaxel
IQR	Interquartile range
OR	Odds ratio
STROBE	Strengthening the Reporting of Observational Studies in Epidemiology
